# Thermal Nociceptive Threshold Testing Detects Altered Sensory Processing in Broiler Chickens with Spontaneous Lameness

**DOI:** 10.1371/journal.pone.0097883

**Published:** 2014-05-21

**Authors:** Becky Hothersall, Gina Caplen, Richard M. A. Parker, Christine J. Nicol, Avril E. Waterman-Pearson, Claire A. Weeks, Joanna C. Murrell

**Affiliations:** School of Veterinary Science, University of Bristol, Bristol, United Kingdom; University of South California, United States of America

## Abstract

Lameness is common in commercially reared broiler chickens but relationships between lameness and pain (and thus bird welfare) have proved complex, partly because lameness is often partially confounded with factors such as bodyweight, sex and pathology. Thermal nociceptive threshold (TNT) testing explores the neural processing of noxious stimuli, and so can contribute to our understanding of pain. Using an acute model of experimentally induced articular pain, we recently demonstrated that TNT was reduced in lame broiler chickens, and was subsequently attenuated by administration of Non-Steroidal Anti-Inflammatory Drugs (NSAIDs). This study extended these findings to a large sample of commercial broilers. It examined factors affecting thermal threshold (Part 1) and the effect of an NSAID drug (meloxicam, 5 mg/kg) and of an opioid (butorphanol; 4 mg/kg) (Part 2). Spontaneously lame and matched non-lame birds (n = 167) from commercial farms were exposed to ramped thermal stimulations via a probe attached to the lateral aspect of the tarsometatarsus. Baseline skin temperature and temperature at which a behavioural avoidance response occurred (threshold) were recorded. In Part 1 bird characteristics influencing threshold were modelled; In Part 2 the effect of subcutaneous administration of meloxicam or butorphanol was investigated. Unexpectedly, after accounting for other influences, lameness *increased* threshold significantly (Part 1). In Part 2, meloxicam affected threshold differentially: it increased further in lame birds and decreased in non-lame birds. No effect of butorphanol was detected. Baseline skin temperature was also consistently a significant predictor of threshold. Overall, lameness significantly influenced threshold after other bird characteristics were taken into account. This, and a differential effect of meloxicam on lame birds, suggests that nociceptive processing may be altered in lame birds, though mechanisms for this require further investigation.

## Introduction

Pain associated with lameness in broiler chickens is poorly understood. Broiler chickens (reared for their meat) reach slaughter weight in around 40 days. Rapid weight gain has repeatedly been associated with the high prevalences of lameness observed in commercial flocks [1], [2]. Alterations in lame birds' behaviour patterns have been observed that minimise walking and standing [3]. Along with reduced mobility in standardised behavioural tests [4], these findings suggest that welfare may be affected by lameness. However, weak correlations between lameness and pathology [5], [6], [7] mean its relationship with pain remains unclear. Non-Steroidal Anti-Inflammatory Drugs (NSAIDs) reduced lame broilers' latency to complete a ‘runway’ mobility tests (carprofen [8]) and altered gait as measured by kinematic analysis (carprofen and meloxicam [9]) but these results could reflect modulation of inflammation without invoking an analgesic effect.

Nociceptive threshold tests involve measuring a behavioural response to a quantified noxious stimulus and are widely used explore the neural processing underlying pain perception in animals. Altered responses to thermal or mechanical stimuli have been documented in farm animal species with production-related, putatively painful conditions such as lameness in sheep [10] and cattle [11], [12]. Chickens have been used extensively in pain research over the last 10 years and are very suitable for this type of quantitative testing. Gentle and colleagues have systematically investigated chicken neurophysiology, and found many similarities between avian and mammalian pain related neurophysiology. The peripheral chicken sensory nervous system contains Aδ and C fibre nociceptors that are indistinguishable, structurally and functionally, from mammalian primary afferent fibers [13], [14], [15], [16]. The response to the external and intradermal administration of inflammatory mediators such as bradykinin, substance P and acetylcholine have been well characterized [17]. For example, following intra-articular injection of sodium urate into the joint in chickens, the joint became inflamed for at least 3 hours showing significant swelling and reddening as well as sensitization of the joint capsule C fibre nociceptors [18], and this was associated with persistent pain related behaviours. Chicken nociceptive and inflammatory responses are also attenuated by opioids and NSAIDs similarly to mammals [19], [20].

We previously developed a method for measuring thermal and mechanical nociceptive thresholds (TNT) in unrestrained broilers [21]. Using this method, we were able to detect thermal (primary) hyperalgesia in an acute model of experimentally induced inflammatory pain, measured as a behavioural response at a lower temperature compared with control-group birds, and its attenuation by NSAIDs [22]. This supports the utility of measuring threshold as a component of pain experience in broilers.

Applying this method to commercially reared birds with and without spontaneous lameness would be a significant step in research into pain processing and thus our understanding of the welfare consequences of lameness for a very large population of human-managed birds. A difficulty is that as well as body mass and growth rate, lameness is associated with various characteristics including age, sex, strain and foot pad dermatitis [1], [2], [23], [24] that might also affect threshold. Indeed, recent studies of thermal and mechanical threshold in young pigs have reported influences of age and mass [25], [26]. Using large, matched samples of birds and modelling the effects of such factors can mitigate this; we have thus demonstrated significant effects of lameness on mobility, irrespective of confounds such as mass [4].

Here, our aim was to use the same approach to compare TNT in spontaneously lame and non-lame birds while accounting for other differences in bird characteristics (Part 1). We also aimed to examine the effects of the NSAID meloxicam, and the opioid drug butorphanol, on TNT (Part 2). Butorphanol increased TNT in healthy horses [27] and altered behaviour in laying hens with bone fractures in a manner suggesting an analgesic effect [28], [29].

We predicted that lame birds would have lower thresholds than non-lame birds (Part 1); that administration of meloxicam would obtund hyperalgesia in lame birds, without altering threshold in non-lame birds (Part 2) and that butorphanol would have an anti-nociceptive effect in all birds (Part 2). Our aims were met but our findings were not consistent with our predictions.

## Methods

### 1. Ethics statement

This study was carried out under Home Office Licence (PPL30/2865) and approved by the University of Bristol Ethical Review Group. The Home Office Code of Recommendations for the Housing of Poultry was met or exceeded at all times. Birds were euthanised by a pre-2013 approved Schedule One method (dislocation of the neck or barbiturate anaesthetic overdose) within three days of final data collection. Additional predetermined humane end-points used in this study were as follows: (i) birds that became excessively lame (>GS 4); (ii) any bird that demonstrated obvious signs of distress or illness.

### 2. Birds

Mixed sex broiler chickens of two strains were acquired from commercial flocks located within South West England at 25–35 days of age. As far as possible, equal numbers of non-lame (Gait Score 0–1) and lame (Gait Score 2.5–4) birds were selected from each farm, using the criteria of Kestin et al [30], and transported to the School of Veterinary Science. Birds were identified using coloured stock marker and housed in groups of 12 in pens measuring 3.05×1.22 m on wood shavings. Animal accommodation was climate-controlled at approximately 20°C and maintained on a 16:8 hour light:dark schedule. Birds had *ad libitum* access to water and commercial feed.

Data presented in Part 1 (the baseline study) and Part 2 (examination of drug treatments) were collected simultaneously as part of a larger study and are reported in two ways: Part 1 describes the findings for all birds administered saline only (*i.e.* birds received no drug treatment) and comprised 167 birds of two strains from 19 flocks. Included within these 167 birds were the 74 birds used as saline control groups for the meloxicam and butorphanol cohorts of Part 2. These birds came from 8 flocks of the same single strain. Thus data from 74 birds are reported in both parts of the experiment. Birds within a flock were used to test only a single antinociceptive drug; in each case half of the birds received saline and half received the drug treatment.

Bird gait score remained stable for only a short period of time following transfer to the research facility; therefore most flocks were kept for four testing days. Some birds were excluded from testing because their gait varied such that they did not fit within our prescribed lame or non-lame gait score ranges on the day of study. For these reasons, a cross-over design could not be employed and all data are unpaired.

#### 2.1 Measurement of bird characteristics

Birds were tested between the ages of 32 and 43 days. On the day before and again on the morning of testing, birds were weighed and gait scored by two experienced researchers. One gently encouraged the bird to walk the length of the pen (without handling or contact) while the other watched from outside the pen. Both wrote down the gait score independently using the method described by Kestin et al [30] but with the inclusion of half-scores. Scores were compared and, for any bird where the researchers were not in full agreement, additional observations of walking were made until the score was agreed. If a score differed by more than 0.5 from the previous day's score, (*e.g.* if a bird changed from GS0.5 to 1.5), the bird was excluded from testing. Birds of GS 0–1 were allocated to the non-lame group and birds of GS 2.5–4 were allocated to the lame group; this formed the binary explanatory variable *lameness*. Individuals allocated a GS outside of this range were not used for data collection. Broilers were also weighed (*mass*), and any *hock burn* and/or *foot pad dermatitis* were recorded using a severity scale of 0–4 [31] but using additional half scores; for each lesion type, birds were assigned a score based on the average of both legs. Sex was determined at post-mortem, carried out within 3 days of completion of testing, at which time the hock joints were dissected and any gross pathology recorded: *injury*, *infection* (*e.g.* swelling/inflammation, excessive or discoloured fluid), and skeletal *deformity*. This information was also combined with observations made during gait scoring to generate a binary score indicating the presence or absence of evidence for leg *pathology*. As swabs of the joints were not taken for microbial analysis, infection type could not be definitively confirmed. On each test day, birds were assigned to lame/non-lame and drug/saline groups matched for age and balanced for weight as far as possible.

### 3. Thermal Nociceptive Threshold Test

The development of this technique is described in detail by Hothersall et al [21]. Briefly, a ramped thermal stimulus was applied (heating rate 1°C/second) via a probe attached to the tarsometatarsus and operated by a hand-held unit (equipment commissioned from TopCat Metrology, Cambridge). Birds were tested in an adjacent room maintained at between 17°C and 20°C, where they were transferred to individual wire cages (38 cm×41 cm×53 cm) with solid foam floors, 20 min prior to testing to allow them to settle and the probes to reach skin temperature. Birds were unrestrained and free to move around the cage; they remained in visual contact with other birds and had *ad libitum* access to food and water throughout testing. Birds were tested on either the left or right leg; choice of leg was balanced as far as possible across gait score and mass. Skin temperature was recorded before heating commenced (baseline) and again when a behavioural response occurred (threshold), at which point heating was immediately stopped. Behavioural responses consisted of the bird suddenly shuffling (moving feet without standing), twitching (body movement only), stamping, stretching a leg, rising to its feet or pecking the probe. Should a bird not respond with a clear behavioural end-point indicating that the nociceptive threshold had been reached, heating was stopped when the probe temperature reached 50°C to prevent skin damage. Most birds were bilaterally lame but any bird demonstrating clear unilateral lameness was tested on the lame leg. The experimenter performing the tests was blinded as to which birds received saline or drugs, but it was not possible to be fully blinded to the lameness category as in many cases this was visually obvious.

### 4. Part 1: Effects of lameness and other bird characteristics on baseline thermal threshold

One hundred and sixty seven birds each underwent a single session of 5 tests without removing the probe. Tests were separated by an interval of at least 10 minutes [22].

### 5. Part 2: Effects of analgesic drugs on thermal thresholds

A total of 142 birds received a subcutaneous treatment of either saline (1.5 mL) or test drug; although the investigators were blinded to treatment allocation (saline or drug), they were aware of which drug was being evaluated at any one time. The meloxicam group received 5 mg/kg of meloxicam (Metacam, 5 mg/mL injectable solution, Boehringer Ingelheim) and the butorphanol group received 4 mg/kg butorphanol tartrate (Torbugesic, 1% W/V injectable solution, Fort Dodge Animal Health Ltd.). The dose of meloxicam was selected on the basis that it obtunded thermal hyperalgesia in a study of induced lameness [22]. The dose of butorphanol was based on previous data from laying hens [28], pharmacokinetic data from broilers [32] and pilot data (unpublished). Testing was as per Part 1 except that it started at a predefined time point after drug or saline administration, based on pharmacokinetic data for butorphanol [32] and meloxicam [33]. A longer test period was used for butorphanol than meloxicam due to a lack of data on the precise time of its bioactivity. Thus for meloxicam, birds underwent 5 tests between 3.5 and 4.5 hours after dosing; for butorphanol, birds underwent 8 tests between 15 minutes and 2 hours after dosing.

### 6. Statistical Analyses

Data from birds in Part 1 and Part 2 were analysed separately. Within Part 2, separate analyses were conducted for each drug treatment.

Descriptive statistics detailing the characteristics of the test cohorts were generated using SPSS Version 19 (Tables 1 and 2). To investigate the effect of lameness and/or drug treatment on thermal threshold, while accounting for other variables that could affect behavioural response, random-intercept nested models were generated using MLwiN v2.25. These models adjusted for non-independence due to clustering within groups. To account for the repeated nociceptive threshold measurements, measurement occasion (*i.e.* test order) (level 1) was nested within individual bird identity (level 2) nested within flocks (level 3), and test order (1–5) was included as a fixed effect (*e.g.* Steele, [34]) meaning that it was treated as a repeated measure. Thermal threshold and baseline skin temperature were analysed as response variables in separate models, with skin temperature included as an explanatory variables (also known as a predictor) in the former case, and leg (left or right) included as a predictor in both. When skin temperature contributed significantly to a model, this was explored further by creating an additional model with skin temperature as the response variable.

**Table 1:** Key sample characteristics for Part 1 test cohort.

Characteristic	Lame	Non-lame	Association/difference
Sex	Male: n = 74; Female: n = 25	Male: n = 18; Female: n = 50	χ^2^ = 38.0, df = 1, p = <0.001
Age^1^	36±4 (32–39)	35±4 (32–43)	z- = −2.0, p = 0.046
Mass^2^, kg	1.92±2.71 (0.97–2.71)	1.55±0.24 (0.87–2.11)	t = 8.1, p<0.001
Hock Burn^1^^,^ 3	0.0±0.5(0–3)	0.0±0.0 (0–3)	z = −3.1, p = 0.002
Foot Pad Dermatitis^1^^,^ 3	0.75±2.0 (0–3)	= 0.0±1.0(0–3)	z = −4.2, p<0.001
Pathology present (%)	31.0	1.5	χ^2^ = 21.5, p<0.001
Of which, type^4^ (%)			
Infection	19.3	100.0	
Deformity	3.2	100.0	
Injury	87.1	0.0	

1 Median ± IQR (range).

2 Mean±SD (range).

3 Where 0 =  none, 4 =  severe open ulcers (Welfare Quality, 2009).

4 In those individuals with an identified pathology the prevalence of each pathological ‘type’ was also calculated. Each type was recorded independently (therefore allowing >100% total).

**Table 2:** Key sample characteristics for birds administered meloxicam or saline (n = 68).

Characteristic	Saline		Meloxicam	
	Lame	Non-lame	Lame	Non-lame
Sex	Male: n = 15 Female: n = 3	Male: n = 7 Female: n = 12	Male: n = 9 Female: n = 9	Male: n = 6 Female: n = 7
Mass^1^ (kg)	1.93±0.28 (1.50–2.39)	1.45±0.20 (1.07–1.82)	1.84±0.33 (1.14–2.64)	1.31±0.32 (0.87–1.95)
Hock Burn^1^^,^ 2	0.5±0.7 (0–2)	0.0±0.1 (0–0.5)	0.7±1.0 (0–3)	0.2±0.2 (0–1)
Foot Pad Dermatitis^1^^,^ 2	1.5±0.9 (0–2.5)	0.6±0.8 (0–1.5)	1.4±1.0 (0–3)	1.0±0.8 (0–2)
Pathology (%)	38.9	4.8	38.9	7.7
Of which, type^3^ (%):				
Infection	0.0	0.0	42.9	0.0
Deformity	100.0	100.0	57.1	100.0
Injury	0.0	0.0	0.0	0.0

1 Mean±SD (range).

2 Value assigned according to a severity scale of 0–4, where 0 =  none, 4 =  severe open ulcers (Welfare Quality, 2009).

3 In those individuals with an identified pathology the prevalence of each pathological ‘type’ was also calculated. Each type was recorded independently (therefore allowing >100% total).

To examine the effects of bird characteristics on thermal threshold, the explanatory variables (*treatment*; *strain*; *age*; *mass*; *hock burn; foot pad dermatitis,* plus binary scores for l*ameness, sex*; and for the various indicators of *pathology*) were initially entered separately into a model (see **Table S1 in File S1**). Significant variables (p<0.05) were then concurrently entered and all other variables re-entered sequentially; any that remained significant were also retained. The combination of predictors that explained the greatest amount of variability was selected as the final model. Interactions between predictors were explored where there was an *a priori* reason to expect a relationship to exist. The significance of individual predictors in a model was tested using Z-tests, whereby the coefficient was divided by the standard error of coefficient to generate respective Z-values. *P*-values were calculated as the area of the normal distribution greater than or equal to the Z-value, multiplied by two (two-tailed analysis). The significance of interactions in a model was tested using χ^2^-tests and the deviance in log-likelihood between models both with and without the interaction. Data were transformed as necessary and standardised residuals were calculated and plotted to ensure that assumptions of normality and homoscedasticity were met. All results refer to the predicted effect of an explanatory variable described by the final model (using the reference values shown in **Table S1 in File S1**), rather than raw data, unless stated otherwise.

## Results

### 1. Part 1: Effects of lameness and other bird characteristics on thermal nociceptive threshold

#### 1.1 Descriptive statistics

A summary of the key characteristics of the cohort is shown in Table 1. Lame (n = 99) and non-lame (n = 68) birds differed significantly in a number of characteristics. Lame birds were slightly but significantly older and heavier than non-lame birds and there was a significant association between sex and lameness. Scores for *hock burn* and *foot pad dermatitis* were both slightly but significantly higher in lame birds (Table 1).

#### 1.2 Relationship between lameness and thermal nociceptive threshold

The statistical model showed that *lameness* was a significant influence on thermal nociceptive threshold (p<0.001). However, contrary to our hypothesis, lame birds had a higher threshold than non-lame birds. Lameness increased threshold by 1.1°C (95% Confidence Interval, CI: 0.5–1.8°C). Keeping the other modelled factors at their reference values (*skin temperature*  =  mean; *test*  = 3) this gave predicted threshold values of 41.3°C for non-lame and 42.4°C for lame birds. See **Table S2 in File S1** for model details.

**Figure 1:**
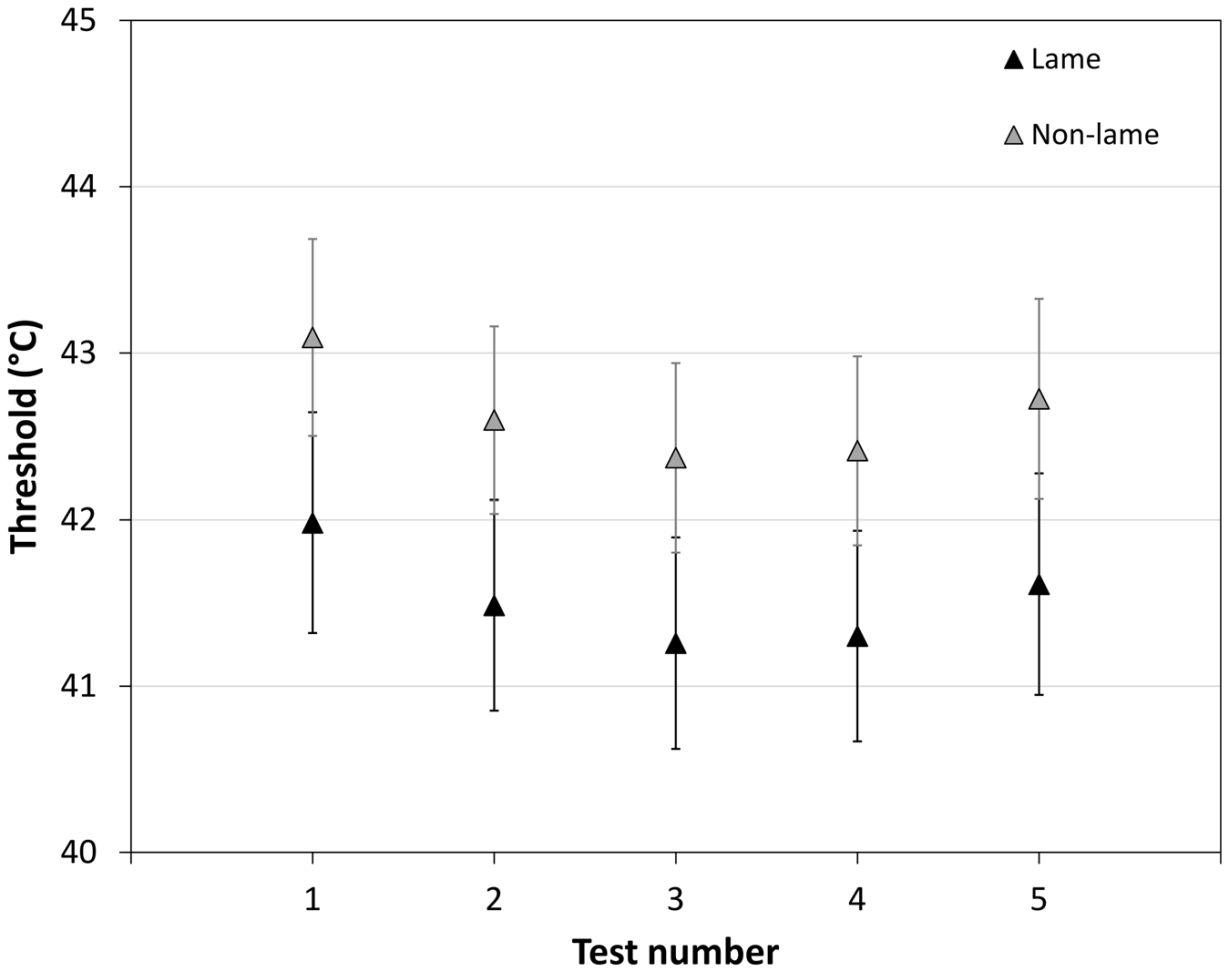
Effect of test number on threshold. For lame and non-lame birds, the modelled predictions (+/−95% CI) of the effect on thermal nociceptive threshold of test number (keeping other factors in the model at their reference values).

This result was unlikely to be an artefact of other influences as these were accounted for in the final model. Other significant influences on thermal threshold were *skin temperature* (p<0.001) and *test number* (p<0.001). None of the other bird characteristics increased the variability explained by the model once lameness, skin temperature and test number were included. *Test number* and *skin temperature* fitted the data best as quadratic terms: threshold decreased slightly between Test 1 and Test 3 before increasing across subsequent tests (Figure 1).

**Figure 2:**
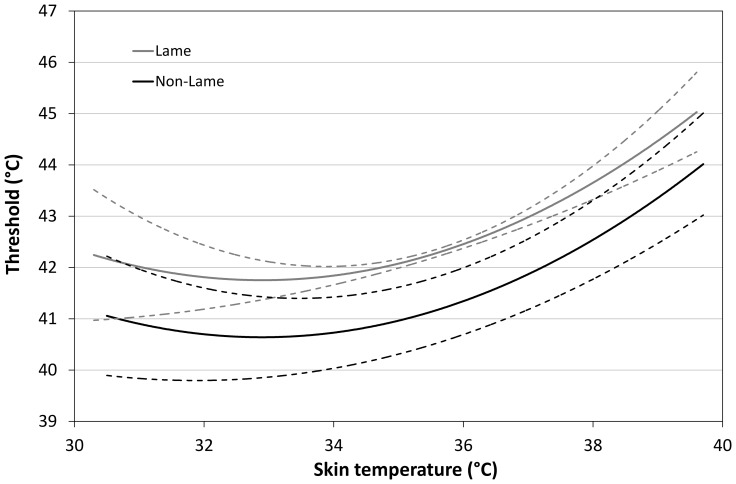
Effect of skin temperature on threshold. For lame and non-lame birds, the modelled predictions (+/−95% CI) of the effect on thermal nociceptive threshold of skin temperature (keeping other factors in the model at their reference values).

Threshold increased with *skin temperature* to describe a tick-shaped curve (Figure 2); it changed relatively little across the lower range of skin temperatures but was increasingly affected at higher temperatures. However, even with all these factors included, the statistical model explained only 14.4% of variation in threshold value.

#### 1.3 Factors affecting baseline skin temperature

Factors affecting baseline skin temperature were modelled due to its significant influence on threshold. *Lameness* was not a significant predictor of skin temperature. The multiple factors that did predict skin temperature together explained only 18.8% of the variation. Skin temperature in birds of *Strain* 1 was 1.3°C (CI: 0.2–2.4°C) lower than in birds of *Strain* 2 (p = 0.02), and was 0.7°C (CI: 0.3–1.2°C) lower in birds with signs of *pathology* (p = 0.003). A 1 kg increase in *mass* increased skin temperature by 0.8°C (CI: 0.2–1.8°C; p = 0.008), and a one-unit increase in *foot pad dermatitis* score increased it by 0.5°C (CI: 0.3–0.8; p<0.001). Skin temperature in birds tested on the right *leg* was 0.4°C (CI: 0.1–0.8) higher than in birds tested on the left *leg* (p = 0.02). *Test number* was again fitted as a quadratic term: skin temperature increased slightly between Test 1 and Test 3 before levelling off over subsequent tests (p<0.001). See **Table S3 in File S1** for model details.

The greatest proportion of unexplained variance remaining after fitting the models was at the individual bird level (thermal nociceptive threshold: 54.1%, skin temperature: 47.2%), with slightly less attributable to tests within birds (thermal nociceptive threshold: 34.6%, skin temperature: 28.7%) and the least to differences amongst flocks (thermal nociceptive threshold: 11.3%, skin temperature: 24.7%).

### 2. Part 2: Effects of analgesic drugs on thermal nociceptive threshold

In Part 2, the models assessed the effect of each drug whilst attempting to account for other potential influences on threshold or skin temperature.

#### 2.1 Meloxicam

The composition of the sample is shown in Table 2.

**Figure 3:**
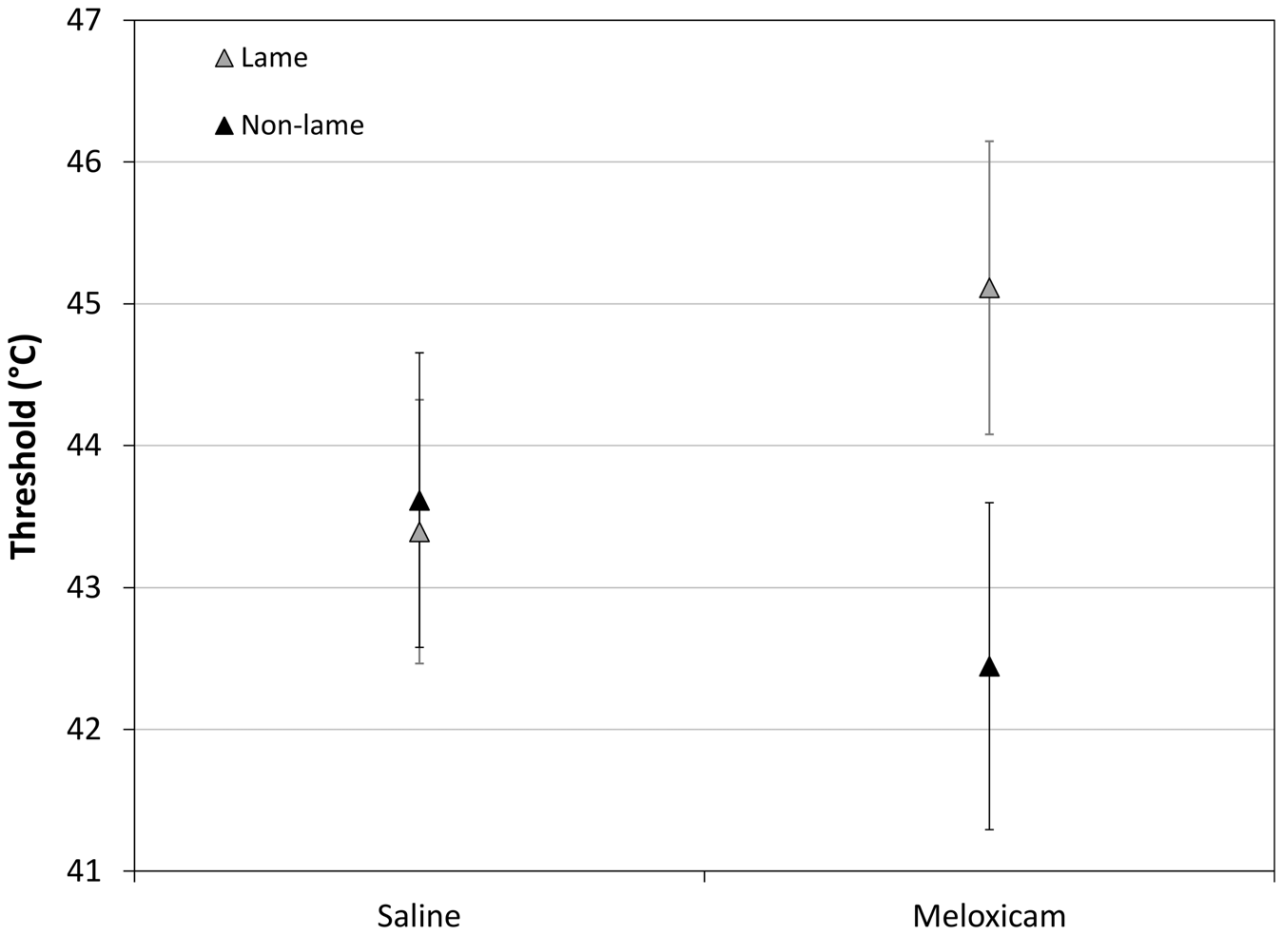
Effects of meloxicam on threshold. Modelled predictions (means ±95% confidence intervals) of thermal nociceptive threshold for lame and non-lame birds administered saline or meloxicam (keeping all other variables in the model at reference values.

Thermal Nociceptive Threshold: the effect of meloxicam on thermal nociceptive threshold differed between lame and non-lame birds (lameness x drug interaction; p<0.005, Figure 3). Meloxicam increased threshold in lame birds, from 43.4°C (CI 42.5–44.3°C) to 45.1°C (CI 44.1–46.1), whereas contrary to expectation it decreased threshold in non-lame birds from 43.6°C (CI 42.6–44.7°C) to 42.4°C (CI 41.3–43.6°C).

Other significant predictors in the model were *test number* (p = 0.02), *sex* (p = 0.009) and *skin temperature* (p<0.001) – details shown in **Table S4 in File S1**. Together with the drug/lameness interaction, this explained 35.3% of the variation in threshold. Flock-level differences were non-significant and flock was removed from the hierarchical structure of the model. After fitting the model, the unexplained variance was divided between the level of test (45.9%) and individual bird (54.1%).

**Figure 4:**
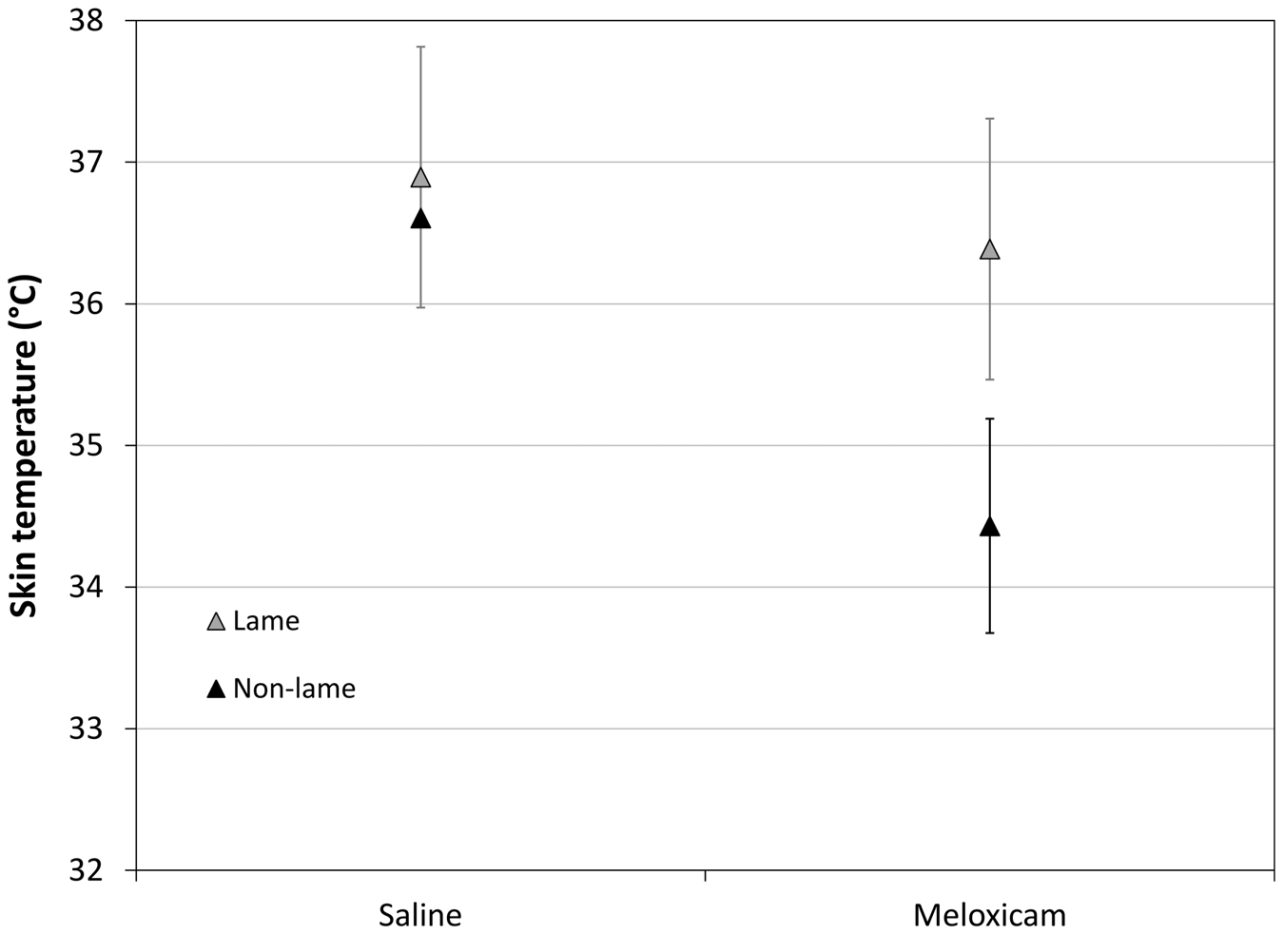
Effects of meloxicam on skin temperature. Modelled predictions (means ±95% confidence intervals) of skin temperature for lame and non-lame birds administered saline or meloxicam (keeping all other variables in the model at reference values).

Skin temperature: as with threshold, meloxicam affected skin temperature differently in lame and non-lame birds (*lameness* x *drug* interaction, p<0.001; Figure 4). Meloxicam decreased skin temperature significantly in non-lame birds, from 36.6°C (CI 36.0–37.2) to 34.4°C (CI 33.7–35.1°C) but not in lame birds (from 36.9°C (CI 35.7–38.1) to 36.4 (CI 35.7–37.1)). The final model explained 16.3% of variation in skin temperature, and the only other significant predictor was *test number* (p<0.001). (see **Table S5 in File S1**). Again, flock was removed from the model structure and after fitting the model, the majority of unexplained variance existed at the level of test (74.6%) rather than individual bird (25.4%).

#### 2.2 Butorphanol

The composition of the sample is shown in Table 3.

**Table 3:** Key sample characteristics for birds administered butorphanol or saline (n = 76).

Characteristic	Saline		Butorphanol	
	Lame	Non-lame	Lame	Non-lame
Sex	Male: n = 16 Female: n = 3	Male: n = 2 Female: n = 15	Male: n = 18 Female: n = 2	Male: n = 8 Female: n = 12
Mass^1^ (kg)	2.05±0.26 (1.66–2.51)	1.73±0.19 (1.49–2.19)	2.16±0.30 (1.61–2.69)	1.74±0.23 (1.33–2.12)
Hock Burn^1^^,^ 2	0.1±0.2 (0–1)	0.1±0.2 (0–0.5)	0.3±0.1 (0–1)	0.1±0.2 (0–1)
Foot Pad Dermatitis^1^^,^ 2	0.6±0.8 (0–2.5)	0.4±0.5 (1.5)	0.6±0.7 (0–1.5)	0.4±0.5 (0–2)
Pathology (%)	36.8	0.0	35.0	0.0
Of which, type^3^ (%):				
Infection	57.1	0.0	57.1	0.0
Deformity	42.9	0.0	42.9	0.0
Injury	0.0	0.0	14.4	0.0

1 Mean±SD (range).

2 Value assigned according to a severity scale of 0–4, where 0 =  none, 4 =  severe open ulcers (Welfare Quality, 2009).

3 In those individuals with an identified pathology the prevalence of each pathological ‘type’ was also calculated. Each type was recorded independently (therefore allowing >100% total).

Thermal nociceptive threshold: contrary to our hypothesis, *drug* was not a significant predictor of thermal threshold; nor was there any significant interaction between *drug* and *lameness*. *Lameness* did increase predicted threshold slightly (<p = 0.001), by 1.1°C (CI: 0.5–1.8°C), from 41.5°C (CI 41.0–42.0°C) to 42.6°C (CI 42.1–34.1°C).

Other significant predictors in the model were *skin temperature* (p<0.001) and *test number* (p<0.001), which together explained 19.3% of variation in threshold. Thermal threshold increased with *skin temperature* in a ‘tick-shaped’ curve similar to that reported in Part 1. Threshold increased slightly with *test number* within the first hour before gradually levelling off over subsequent tests. Model details are shown in **Table S6 in File S1**. Again, flock was removed from the model structure and after fitting the model, the unexplained variance was approximately equally divided between the level of test (47.0%) and individual bird (53.0%). Similar results were obtained for the single hour data sub-sets. Skin temperature was modelled, but neither *lameness* nor *drug* had any significant effect on skin temperature, hence these models are not reported here.

## Discussion

This is the first study to report TNTs in a large population of birds with spontaneous lameness and provides evidence of altered nociceptive processing in lame birds. Contrary to our initial predictions, in Part 1 we found that lame birds' thresholds were significantly higher than those of non-lame birds. In Part 2, we predicted that lame birds' thresholds would be normalised by the NSAID drug meloxicam, and that threshold would be elevated in both lame and non-lame birds by the opioid butorphanol tartrate. We did observe a differential effect of meloxicam, but in fact threshold was *higher* in lame birds and *lower* in non-lame birds administered meloxicam compared with their counterparts administered saline. No significant effect of butorphanol was observed on either group.

Our modelling approach means we can be confident that these findings are not simply an artefact of partial confounds with bird characteristics. Although distributions of mass and sex were partially confounded with lameness, lameness was retained in the final model in all cases. In contrast, with the exception of sex in the meloxicam group, bird characteristics and pathological status were not significant predictors of threshold, after accounting for lameness. This is consistent with our recent findings that performance on two standardised tests of mobility was most consistently predicted by lameness rather than other characteristics [4]. Together, these findings suggest that there is a component of lameness (beyond, *e.g.*, being heavy and male) that influences mobility and nociceptive processing and may represent pain or discomfort.

The mean values recorded in Part 1 were consistent with our previous published data, in that mean thresholds for non-lame birds were slightly lower but with overlapping standard deviations (*e.g.* mean/SD 42.5±2.5°C [21]; 43.7±1.8°C [22]. Some of this variability is likely to be attributable to differences in age and/or mass. The values are at the lower end of the range (39–61°C) identified by Gentle and colleagues [14] for polymodal C-fibre nociceptors in the tarsometatarsus of the hen, using a radiant heat source. The use in the present study of a thermode in contact with the skin is typical of thermal nociceptive threshold testing in live animals and may result in lower thresholds partly because the thermode also directly activates low threshold mechanoreceptors.

### 1. Effects of lameness on thermal nociceptive threshold

The finding, in Part 1, that lame birds had a higher TNT than non-lame birds, was unexpected. In most published studies in other species, and in broilers with experimentally induced arthropathy [22], lameness was associated with a decrease in nociceptive thresholds. In the latter study, the thermal probe was placed adjacent to the inflamed joint and so measured primary thermal hyperalgesia that is indicative of peripheral sensitization [35] – *i.e.* increased sensitivity at the site of injury. The hypoalgesia detected in the lame birds in the present study is difficult to interpret, partly because sampling birds with commercially relevant lameness meant that the aetiology of their lameness was unknown and could not be definitively attributed to pathology in the intertarsal (hock) joint. It is possible that that the thermal device was positioned remote to the site of tissue injury, in the secondary area.

Secondary thermal hyperalgesia (a reduction in threshold remote from the injured area) is not reported to occur with thermal stimuli that selectively activate C fibre nociceptors [35], [36], [37], which are the nociceptor type that would be activated by the heating speeds used in this study. One explanation could be altered sensory processing of thermal stimuli due to small fibre neuropathy [38]. Secondary thermal hypoalgesia (increased threshold) has also been reported in dogs with osteoarthritis [39]; however, the likelihood of broilers developing such pathologies during their very short lifespan seems low. Reduced responsiveness to thermal [40] and mechanical [41] stimulation has been observed in dairy cows with mastitis but could be ascribed to artefacts such as order effects or reluctance to move. Neither of these explanations would account for our findings as almost none of the behavioural endpoints required weight-bearing. Furthermore, reluctance to move or bear weight on the affected limb was not seen in our earlier study, where induced arthropathy resulted in hyperalgesia (decreased threshold) [22].

Di Giminiani et al [26] recently reported lower TNTs to CO_2_ laser stimulation in small (30 kg) versus large (60 kg) juvenile pigs. Mass was not a significant predictor of threshold in our sample, even in preliminary univariate analyses. Basal skin temperature was a significant predictor of threshold and was itself affected by a complex set of factors including mass; inclusion of skin temperature in our threshold model means that threshold differences cannot be attributed to mass-mediated group differences in skin temperature. By sampling lame and non-lame birds from the same flocks, we were able to largely match other factors such as age that might influence skin thickness or conductivity and age was not significant in any of our models.

An alternative explanation is that a stress response to chronic pain associated with lameness may have reduced lame birds' responsiveness to temperature, resulting in higher TNTs. The mechanism for this is unclear, but a recent study in pigs also documented ‘baseline’ hypoalgesic responses to thermal stimuli (*i.e.* not to acute induced pathology) in pre-natally stressed juvenile pigs - they exhibited reduced responsiveness (shorter response durations) to cold stimulation of the tail root [42].

Although small, the difference in threshold between lame and non-lame birds was repeatedly observed throughout the study during preliminary analyses of individual batches of birds. This supports the biological (as well as statistical) significance of this result. Matching the groups as closely as possible within a commercial population and including other bird characteristics in our models enabled us to detect these relatively subtle differences despite variability.

### 2. Effects of analgesic drugs on thermal thresholds

We originally anticipated that in Part 2, the difference in TNT between lame and non-lame birds would be reversed or attenuated by meloxicam. A differential response was in fact observed in Part 2, represented by the significant drug by lameness interaction, but threshold was actually *higher* in lame birds and *lower* in non-lame birds given meloxicam compared with their control (saline) counterparts. Thresholds for the control groups were slightly higher than in Part 1 and the baseline difference between lame and non-lame groups was numerically but not significantly higher. Given that these birds were a sub-set of the sample in Part 1, this may be due to lower statistical power in the smaller group sizes. Skin temperature was modelled as it was again a significant predictor of threshold. Here too, a significant drug by lameness interaction was observed: meloxicam lowered skin temperature in non-lame birds only. NSAIDs have previously been recorded to lower cloacal temperature in birds [43], although the underlying mechanism is unknown. The effect of meloxicam on threshold was not an artefact of skin temperature as this was included in the threshold model. The two findings are consistent with a differential effect of meloxicam in lame and non-lame birds.

There was no effect of butorphanol (4 mg/kg) on threshold or skin temperature. In mammals, butorphanol tartrate increased TNT in foals [27] and cats [44]. This suggests that we did not identify an appropriate combination of dose, route of administration and timing. Butorphanol at 2 mg/kg was sufficient to induce place preference in laying hens with keel fractures but not in healthy hens [29], indicating an analgesic rather than hedonic action. Bioavailability and drug clearance rate can vary considerably amongst bird species [33] and may also vary between highly selected sub-species such as laying and broiler chickens. This result was unexpected as our (unpublished) data showed that spontaneous activity in broilers was reduced in the two hours following administration of this dose of butorphanol.

### 3. Other significant predictors of threshold or skin temperature

Skin temperature and test number had consistent, significant effects on threshold. Including these within our models allowed us to account for their influence rather than, for example, assuming a direct relationship by calculating threshold as excursion from baseline skin temperature. For example, the relationship between skin temperature and threshold was not detected by tests of correlation in our earlier method paper [21]. In that paper we discussed reasons for the influence of test number on skin temperature – despite a settling period between fitting probes and testing – that were replicated in this study.

In contrast, none of the bird characteristics we measured were consistent predictors of threshold. We attempted to address possible heterogeneity of lameness by assigning descriptive pathology ‘types’ (inflammation; deformity; infection; any of these) but none were retained in the models. Combined with the lack of effect of lameness on skin temperature, this further suggests that our results reflect secondary rather than primary hypoalgesia.

Finally, the models were able to explain only a modest proportion (between 14 and 35%) of variability in threshold. The remaining, or random, variability was consistently highest at the level of the individual bird and slightly lower amongst tests (within birds). Individual variability would ideally be addressed by using a cross-over design but this was not feasible given the rapid changes in broilers' gait score. Variability due to differences between flocks was small or non-significant, indicating that sampling from multiple farms was a valuable way to obtain a representative sample of lame and non-lame birds.

### 4. Methodological note

Thermal nociceptive threshold testing widely employs skin temperature at the time of behavioural response (threshold) as a proxy measure for temperature at the level of the C fiber nociceptors; the latter will undoubtedly be lower due to the insulating nature of skin [45]. The validity of this proxy assumes that the factors affecting heat transfer between the skin and nociceptor are constant between non-lame birds and lame birds (whose skin may be affected by inflammatory processes). We are not aware of any literature contradicting this assumption, but in the absence of a non-invasive technique for measuring core temperature it could not be fully tested.

## Conclusions

Spontaneous lameness significantly influenced thermal nociceptive threshold in broiler chickens. Unexpectedly, threshold was higher in lame than non-lame birds. The NSAID meloxicam had a differential effect on both threshold and skin temperature in lame birds; together the results suggest that lameness may alter nociceptive processing, though mechanisms for this require further investigation. A multi-level modelling approach that took account of potentially confounding bird characteristics proved particularly valuable in studying a commercially relevant population, where subjects are obtained from multiple flocks and capacity to balance groups is limited.

## Supporting Information

File S1 Table S1, Reference values for significant predictors. Table S2, Factors affecting thermal threshold in broilers. Table S3, Factors affecting skin temperature in broilers. Table S4, Factors affecting thermal nociceptive threshold in broilers administered saline or meloxicam. Table S5, Factors affecting skin temperature in broilers administered saline or meloxicam. Table S6, Factors affecting thermal nociceptive threshold in broilers administered saline or butorphanol.
(DOCX)
