# Supplementary material for: Thermal Nociceptive Threshold Testing Detects Altered Sensory Processing in Broiler Chickens with Spontaneous Lameness
Source: PLoS One. 2014 May 21;9(5):e97883. doi: 10.1371/journal.pone.0097883 (PMC4029894; doi:10.1371/journal.pone.0097883)
Supplement: File S1 — Table S1, Reference values for significant predictors. Table S2, Factors affecting thermal threshold in broilers. Table S3, Factors affecting skin temperature in broilers. Table S4, Factors affecting thermal nociceptive threshold in broilers administered saline or meloxicam. Table S5, Factors affecting skin temperature in broilers administered saline or meloxicam. Table S6, Factors affecting thermal nociceptive threshold in broilers administered saline or butorphanol. (DOCX) [file pone.0097883.s001.docx]

**Table S1: Reference values for significant predictors.**

| **Variable** | **Reference value** |
| --- | --- |
| *Foot pad dermatitis* | 0 (no damage) |
| *Hock burn* | 0 (no damage) |
| *Lameness* | 0 (non-lame) |
| *Leg* | Left |
| *Mass* | Mean |
| *Pathology and pathology sub-categories* | 0 (absent) |
| *Sex* | Male |
| *Skin temperature* | Mean |
| *Strain* | 1 |
| *Test* | Mean (3) |

**Table S2: Factors affecting thermal threshold in broilers.**

| **Variable** | **Coefficient** | **SE (Coeff)** | **z-value** | **χ^2^** | **d.f.** | **p** |
| --- | --- | --- | --- | --- | --- | --- |
| Constant | 42.379 | 0.290 | 146.135 |  |  | <0.001 |
| Lameness | -1.113 | 0.335 | -3.322 |  |  | <0.001 |
| Skin |  |  |  | 50.722 | 2 | <0.001 |
| Test |  |  |  | 21.350 | 2 | <0.001 |

**Table S3: Factors affecting skin temperature in broilers.**

| **Variable** | **Coefficient** | **SE (Coeff)** | **z-value** | **χ^2^** | **d.f.** | **p** |
| --- | --- | --- | --- | --- | --- | --- |
| Constant | 34.289 | 0.553 | 62.005 |  |  | <0.001 |
| *Foot pad dermatitis* | 0.501 | 0.133 | 3.767 |  |  | < 0.001 |
| *Pathology* | -0.741 | 0.248 | -2.988 |  |  | 0.003 |
| *Mass* | -0.792 | 0.301 | -2.631 |  |  | 0.008 |
| *Leg* | 0.436 | 0.182 | 2.396 |  |  | 0.02 |
| *Strain* | 1.308 | 0.573 | 2.283 |  |  | 0.02 |
| *Test* |  |  |  | 181.389 | 2 | <0.001 |

**Table S4: Factors affecting thermal nociceptive threshold in broilers administered saline or meloxicam.**

| **Variable** | **Coefficient** | **SE (Coeff)** | **z-value** | **χ^2^** | **d.f.** | **p** |
| --- | --- | --- | --- | --- | --- | --- |
| Constant | 43.616 | 0.529 | 82.450 |  |  | <0.001 |
| *Skin temperature^1^* | 0.469 | 0.095 | 4.937 |  |  | <0.001 |
| *Sex^2^* | -1.338 | 0.513 | -2.608 |  |  | 0.009 |
| *Test number^3^* | -0.167 | 0.069 | -2.420 |  |  | 0.02 |
| Interaction: *Lameness* x *treatment* |  |  |  | 21.350 | 3 | 0.005 |

^1^Threshold increased by 0.47˚C (CI 0.28-0.66˚C) for each 1˚C increase in skin temperature.
^2^Male birds had a mean threshold 1.3˚C (CI 0.30-2.30˚C) higher than females.
^3^Threshold decreased by 0.17˚C (CI 0.03-0.30˚C) with test number across a session.

**Table S5: Factors affecting skin temperature in broilers administered saline or meloxicam.**

| **Variable** | **Coefficient** | **SE (Coeff)** | **z-value** | **χ^2^** | **d.f.** | **p** |
| --- | --- | --- | --- | --- | --- | --- |
| Constant | 36.606 | 0.315 | 116.210 |  |  | <0.001 |
| *Test number^1^* |  |  |  | 63.593 | 2 | <0.001 |
| Interaction: *Lameness* x *treatment* |  |  |  | 36.825 | 3 | <0.001 |

^1^Fitted as a quadratic relationship: skin temperature initially rose slightly over sequential tests then levelled off across the last two tests.

**Table S6: Factors affecting thermal nociceptive threshold in broilers administered saline or butorphanol.**

| **Variable** | **Coefficient** | **SE (Coeff)** | **z-value** | **χ^2^** | **d.f.** | **p** |
| --- | --- | --- | --- | --- | --- | --- |
| Constant | 131.234 | 35.211 | 3.727 |  |  | <0.001 |
| *Lameness* | 1.135 | 0.349 | 3.252 |  |  | <0.001 |
| *Skin temperature* |  |  |  | 21.001 | 2 | <0.001 |
| *Test number* |  |  |  | 61.687 | 2 | <0.001 |
